# Is it me or not me? Modulation of perceptual-motor awareness and visuomotor performance by mindfulness meditation

**DOI:** 10.1186/1471-2202-13-88

**Published:** 2012-07-30

**Authors:** José Raúl Naranjo, Stefan Schmidt

**Affiliations:** 1 Department of Psychosomatic Medicine and Department of Environmental Health Sciences, University Medical Center Freiburg, Breisacherstr. 115b, 79106, Freiburg, Germany; 2 Institute for Transcultural Health Studies, European University Viadrina, Frankfurt (Oder), Germany; 3 Samueli Institute, Brain, Mind and Healing Program, Alexandria, VA, USA

**Keywords:** Self-agency, Perceptual-motor awareness, Speed/accuracy trade-off, Attention, Mindfulness meditation

## Abstract

**Background:**

Attribution of agency involves the ability to distinguish our own actions and their sensory consequences which are self-generated from those generated by external agents. There are several pathological cases in which motor awareness is dramatically impaired. On the other hand, awareness-enhancement practices like tai-chi and yoga are shown to improve perceptual-motor awareness. Meditation is known to have positive impacts on perception, attention and consciousness itself, but it is still unclear how meditation changes sensorimotor integration processes and awareness of action. The aim of this study was to investigate how visuomotor performance and self-agency is modulated by mindfulness meditation. This was done by studying meditators’ performance during a conflicting reaching task, where the congruency between actions and their consequences is gradually altered. This task was presented to novices in meditation before and after an intensive 8 weeks mindfulness meditation training (MBSR). The data of this sample was compared to a group of long-term meditators and a group of healthy non-meditators.

**Results:**

Mindfulness resulted in a significant improvement in motor control during perceptual-motor conflict in both groups. Novices in mindfulness demonstrated a strongly increased sensitivity to detect external perturbation after the MBSR intervention. Both mindfulness groups demonstrated a speed/accuracy trade-off in comparison to their respective controls. This resulted in slower and more accurate movements.

**Conclusions:**

Our results suggest that mindfulness meditation practice is associated with slower body movements which in turn may lead to an increase in monitoring of body states and optimized re-adjustment of movement trajectory, and consequently to better motor performance. This extended conscious monitoring of perceptual and motor cues may explain how, while dealing with perceptual-motor conflict, improvement in motor control goes beyond the mere increase of movement time. The reduction of detection threshold in the MBSR group is also likely due to the enhanced monitoring of these processes. Our findings confirmed our assumptions about the positive effect of mindfulness on perceptual-motor integration processes.

## Background

Action has become a central topic in the scientific study of self-awareness. The ability to experience oneself as the cause of an action seems to be a fundamental building block supporting the sense of self. In particular *awareness of action* and the *attribution of agency* are key issues in the neuroscientific study of consciousness [1].

Attribution of agency involves the ability to distinguish one’s own actions and their sensory consequences from those generated by another source. Efferent and afferent information jointly constitute the core of our bodily self-awareness [2]. Efference is a key function of the motor system, responsible for motor control, motor learning, motor prediction and motor correction [3]. Afference, and especially proprioception, provides us with the specific content of our bodily self-awareness [2]. However, afference can be the result of either self-generated actions or externally-generated sensory stimulation. Although we are normally aware of our intention to move as well as of the goal of our movements, we do not have conscious access to all our motor commands and every fine adjustment made to a movement. Nevertheless, high-precision motor control is still possible, relying on internal representations of the actual, desired, and predicted states of our body and the environment [4]. Certain components of these internal representations may become available to awareness when the discrepancy between the predicted and the actual sensory consequences of an action is large [5,6]. The exact threshold above which this perceptual-motor conflict becomes available to awareness is currently a focus of intensive research (for reviews see [4,7,8]).

Under normal conditions, healthy subjects have only a limited awareness of their motor performance [9]. Furthermore, there are pathological cases such as patients with prefrontal lesions [10,11], deafferented patients [12], and schizophrenic patients [13], in which motor awareness is severely impaired. Impairment of self-agency and motor awareness in these patients was studied using a reaching movement paradigm, where a conflict was introduced between the actual movement of the hand and the feedback which is provided. The hand movement consisted on tracing an imaginary line between a starting point and a target on a horizontal surface. The participants’ view on their moving hand was covered to avoid direct feedback. At the same time the participants reaching trajectory was projected onto a second surface mounted directly above the movement area. A perceptual-motor conflict was introduced by providing the participants with a false visual feedback, i.e. an angular deviation of the projected trajectory. Results of these studies suggested that there are two different processes related to solving such a visuomotor conflict. For small deviations, all participants implicitly adjusted their hand trajectory to reach the target, without being aware of the process. For larger angles (∼10°) healthy participants became aware of the conflict between the perceptual and motor process, and switched to a conscious monitoring strategy [9]. In contrast, patients with impaired motor awareness remained unaware of the mismatch and therefore, had more difficulties to compensate for the deviation [10-13].

Previous neuroimaging and neurophysiological studies have assessed the neural basis of perceptual-motor awareness and self-agency. They suggest that the cerebellum [14], the parietal cortex [15], the angular gyrus [16], the insular cortex [16,17], and the prefrontal cortex [11] are involved in signaling the sensory discrepancy between the predicted and the actual sensory consequences of our movements. Overall, these studies have shed light on the behavioral and neurophysiological correlates of awareness and control of actions, and have contributed to understand how abnormalities in the awareness of actions arise in several pathologies (e.g. [8]).

However, only a few studies have assessed whether it is possible to *improve* perceptual-motor awareness by interventions and techniques known to enhance self-awareness. One of the most prominent techniques in this sense is meditation.

One major component in most meditation techniques is the continuous monitoring of present experience which is especially true for mindfulness meditation. In this meditation technique Buddhist practitioners aim towards expanding their attention to all available inputs (sensory, bodily or mental) within consciousness in the present moment. The goal is to maintain a continuous state of moment-to-moment awareness encompassing the full experience of the present moment with certain attitudinal qualities [18,19]. Based on the concept of mindfulness, Jon Kabat-Zinn created a secular eight-weeks behavioral intervention program named *Mindfulness Based Stress Reduction (MBSR)*[20]*,* which teaches not only being mindful within a meditation exercise, but also during everyday activities such as eating, washing, etc. [21]. Therefore mindfulness practitioners are trained in the conscious execution and continuous awareness of body movements, e.g. when reaching out for a certain object. Besides, some converging evidences support that meditation, and specifically an MBSR intervention results in changes in self-related processes. Farb et al. [22] suggested that there are two different forms of self-reference: one relating only to present moment experiences, and another linking experiences with the self-concept across time in a narrative way. They demonstrated in an fMRI study that MBSR results in pronounced brain activity in a specific neural network (comprising the lateral PFC, insula, secondary somatosensory cortex and inferior parietal cortex) underlying a present moment form of self-awareness. This network overlaps with brain areas known to be specifically activated in relation to the experience of self-agency and perceptual-motor awareness (see above). This ‘neural sharing’ gives tentative support to the hypothesis that mindfulness meditation may influence perceptual-motor integration processes and self-agency.

Due to a likely suspension of many mental processes during meditation, larger cognitive resources are available within a meditation state to attend only present time processes [23]. In fact, meditation has a positive impact on several cognitive processes such as emotion regulation [24,25], increasing attention [26,27], pain processing [28,29] and perceptual rivalry [30]. Next to these reports, studies using MRI have demonstrated the capacity of meditation to result in neuroplasticity mechanisms in the central nervous system, which may lead to positive effects [31-34]. Strikingly, only a few scientific studies have specifically addressed the influence of meditation practice on sensorimotor performance so far.

Telles et al. [35] showed significant better visuomotor performance of yoga meditators compared to controls. These results were interpreted as a sign of plasticity in motor control systems associated to the practice of yoga meditation. Jedrczak et al. [36] showed that the number of months of practice of the Transcendental Meditation (TM) significantly predicted higher performance on perceptual-motor speed tests. In contrast to these positive results, other studies have shown that TM is not associated with acquisition of fine perceptual-motor skills [37,38] and with learning and performance of a novel perceptual-motor task [39].

Regarding the possible impact of meditation on perceptual-motor awareness Ranii and Rao [40] showed that after 3 months of yoga meditation, participants reported greater awareness of bodily processes (i.e. bodily sensations resulting from mindful body movements through a series of poses) compared to controls.

The research body on the effects of yoga and TM on motor behavior has led to mixed results, and it is difficult to extract a clear-cut message. Mindfulness is claimed to cultivate a mental stance where internal (somatic and propioceptive aspects of the movement) and external (sensory feedbacks from movements) signals are equally perceived in a balanced way. Based on this claim, we hypothesized that mindfulness practice is an appropriate candidate to assess the impact of meditation on motor behavior.

The objective of the present study was to assess the impact of mindfulness meditation on perceptual-motor awareness, motor accuracy and movement duration in a visuomotor reaching task with false feedback as described above. This was assessed in two different comparisons. First, we compared a group of participants in the MBSR program with an age and sex-matched control group receiving no intervention. Second, we studied the performance of long-term meditators in comparison with a group with no prior experience in meditation. Our results suggest that mindfulness meditation practice is associated with slower body movements, better motor performance, and enhanced awareness of perceptual-motor conflict.

## Methods

### Design

The study presented here consists of two parts, one with a longitudinal and one with a cross sectional design.

In the first part we investigated the effects of a mindfulness based intervention (MBSR) compared to a sex and age matched control group in a *longitudinal* comparison. The participants (short term meditators = SM) in the intervention group were assessed before (t1) and after the eight week program (t2). The control group (CG) was also assessed twice within 8 weeks but did not receive any intervention.

In the second part, we studied the long-term effects of meditation by comparing a group of participants with a long-standing meditation experience (LM) with respect to a group of naïve subjects (NM) in a cross sectional design. In this design, data from participants in both groups LM and NM were compared at one single time point only, when participants in both groups performed the task for the first time. This is in contrast to a longitudinal design with more than one measurement point.

### Participants

Overall 31 participants were recruited. All participants were right-handed and did not have any longstanding experience in techniques impacting on fine-grained motor skills, such as e.g. sewing, craftwork, playing an instrument or Thai Chi. Furthermore, reduced visual acuity, impairment of arm movement and a history of mental illness served as exclusion criteria. The long-term meditator group (LM, N = 9, 5 females) consisted of four monks and nuns of the Buddhist Theravada tradition recruited from monasteries in Germany, Switzerland, and Burma, as well as of five mindfulness meditation teachers. They had an average age of 49.7 years (SD = 7.50) and a meditation experience of 22.0 years (SD = 7.92). The short-term meditator group (SM, N = 11, 9 females) was recruited amongst participants who had already enrolled for a MBSR program. Participation in the study was honored by giving a reduction to the costs of the MBSR program. All participants in this group had no prior experience with meditation. They had an average age of 40.2 (SD =11.44) years. The control group (CG) (N = 11, 10 female) was recruited by public announcement and through personal contacts and had also no prior meditation experience. They were remunerated for their participation. The CG group was recruited in a way to match the self-selected SM group by sex and age. However due to a communication error one male participant was matched with a female control subject of the same age. Mean age of the CG group was also 40.2 (SD = 10.81) years. The group of non-meditators (NM, N = 11) to be compared with the long-term meditators in cross-sectional part was formed from the participants in the SM group by taking their data at t1 (i.e. before the intervention). All participants gave their informed consent. The experiment was approved by the University Medical Center’s ethic commission.

### Intervention

Mindfulness Based Stress Reduction (MBSR) is a structured 8 weeks group program with a group size of not more than 12 participants. Single weekly sessions are 2.5 h in duration, and there is an additional single all-day session per course on a weekend day. Each session covers particular exercises and topics that are examined within the context of mindfulness. These include different forms of mindfulness meditation practice, mindful awareness during yoga postures and mindfulness during stressful situations and social interactions. Because development of mindfulness is predicated upon regular and repeated practice, participants are asked to carry out daily 45-min homework assignments primarily in the form of meditation practice, mindful yoga and applying mindfulness to situations in everyday life. Participants were asked to keep a diary of their daily homework in order to document their efforts.

### Measures

Participants were asked to fill in the Freiburg Mindfulness Inventory FMI [41]. SM and CG participants filled in the questionnaire at t1 and t2. The FMI is a 14-item instrument assessing self-attribution of mindfulness, with higher values standing for a higher degree of mindfulness.

### Experimental apparatus

**Figure 1:**
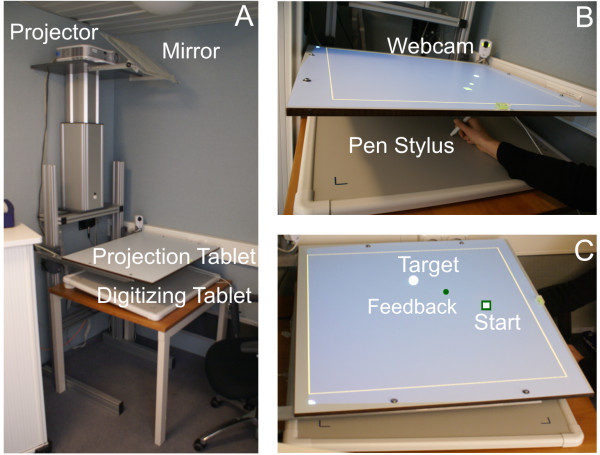
**Experimental setup. A**) The experimental setup for the visuomotor reaching task. **B**) Separation of drawing surface (DT bottom) and visual feedback (PT, top). Movement trajectories are digitized by a pen stylus on DT. Direct feedback of the moving arm is prevented by PT. The experiment was continually monitored by a webcam. **C**) Visual feedback of the participant’s movement from ‘Start’ to ‘Target’, which is represented by a moving green dot on PT.

The experimental apparatus consisted of a 50 cm × 60 cm digitizing tablet (DT, Accugrid Tablet A4, Numonics, USA, 2006) placed on a table and was connected to a computer. Any movement on the DT, was projected by a video projector through a mirror onto a horizontal surface termed projection tablet (PT) just above DT (*cf.* Figure 1A). The participants sat comfortably on a chair and held a pen stylus, so that they could draw on DT. The right hand was placed between both tablets DT and PT, so that the hand and the lower arm was covered in order to avoid direct visual feedback of the own movement (*cf.* Figure 1B). Furthermore the experimental sessions were run in complete darkness in order to prevent from visual cues from the surrounding. When tracing a line on DT the participants could only see as visual feedback a moving green dot (*cf.* Figure 1C). In order to provide a false feedback the output of the DT was processed by the computer using a simple algorithm for adding a linear angular deviation towards the right or the left on the ‘projected’ movement direction.

The digitizing tablet had a high sub-millimetric spatial resolution and also a good temporal resolution (∼10 ms) and allowed reconstructing the kinematics of reaching movements with very good accuracy. The estimated time delay between the motor recording and the visual display on the projection tablet was below 8 ms. The experiment was run by software written by a professional programmer especially for this set-up. This software controlled the random presentation of the target, recorded all timings and movements, and presented a rating scale to the participant after each trial.

### Procedure

Participants were first asked to place the pen stylus on a starting position on the tablet which is close to the body midline and which was represented in the projection tablet (PT) as placing a small red circle within a green square. Our design included EEG measurements across all experimental conditions. The EEG data will be reported elsewhere. Because eyes movements may lead to strong EOG artifacts in the EEG signal, participants were requested to focus their attention to a previously specified region (in the right hemifield) of the tablet, where the target was expected to appear. In this way eye movements (blinks and saccades) were mostly avoided. Because of this constraint, participants were requested to follow with peripheral vision the visual feedback (moving green dot) of their movements.

The start of each trial was indicated by turning green the red circle. After a random baseline period (ranging from 4 to 7 sec.), the visual target appeared at a distance of 200 mm from the starting position. This baseline period between trials was important to assure arm muscle relaxation, in order to avoid contamination of EMG artifacts originated by full extension of the arm in the previous trial, readjustment of head position, as well as eye blinking, etc.

**Figure 2:**
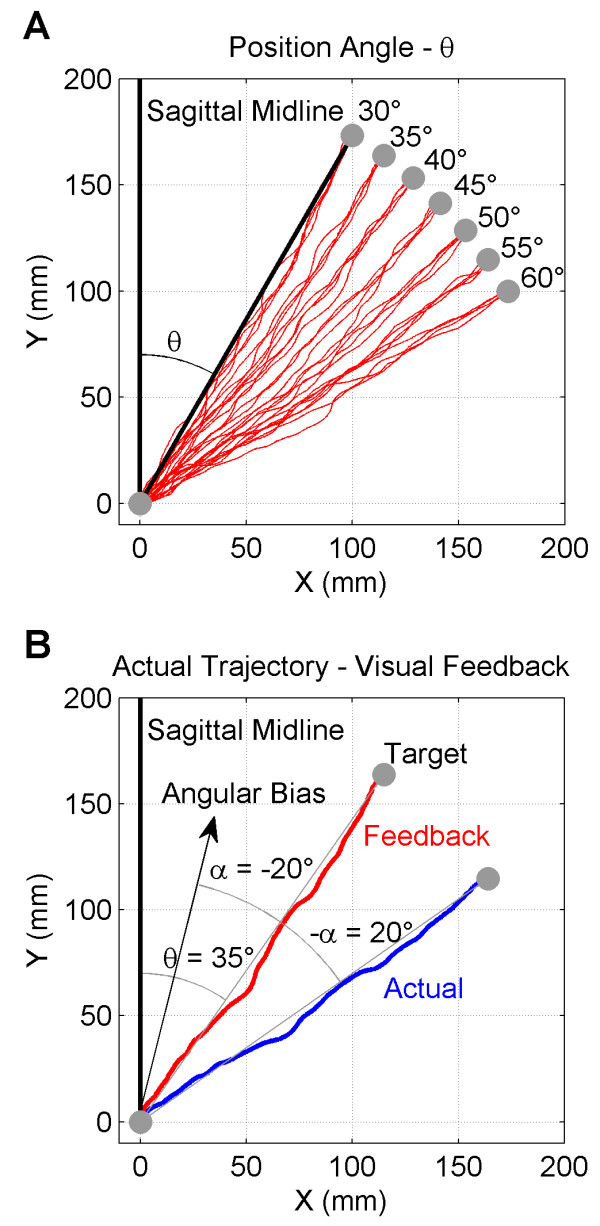
**Representation of 2D movement trajectories. A**) Targets appeared at 7 position angles (*θ*). An example of reaching movements towards the 7 target locations are represented as red trajectories. **B**) Example of an angular deviation to the left (*α* = −20°). Participants should deviate their actual movements (blue) 20° to the right, in order to properly reach the target along the red trajectory given as visual feedback.

The target position angle was randomized across trials (*θ: 30°, 35°,40°, 45°, 50°, 55°, 60°*) with respect to the sagittal midline (*cf.* Figure 2A). Participants were instructed to reach the target, by drawing continuously a straight line between the starting point and the target. Participants were asked to move their hand in a mindful way and at a moderate speed to ensure accuracy, i.e. to reach the target and at the same time to avoid deviations from the straight line. Movement duration was constrained to a 3 s −15 s time frame in order to avoid very fast movements with low accuracy and non-natural very slow movements. The lower threshold was empirically determined in pilot trials.

An angular deviation (*α*) was applied to feedback display in order to create a false feedback of the movement. The bias and its direction (towards the left or right) was randomly assigned from trial to trial, ranging from −27° to 27° in steps of 1° (see Figure 2B for an example). In order to correct for this bias participants had to move the pen on DT along a trajectory, that is deviated in the opposite direction to the bias. By virtue of this strategy, participants may immediately recalibrate their movement and reach the target displayed on PT. At the end of the trial, participants were asked to report their subjective perception of the size of a perceptual-motor conflict between the projected movement and the real movement on a stepwise scale from 0 to 5. Participants were instructed to report *SR* = 0 only if they perceived no *external* perturbation and no perceptual-motor conflict, felt in full control of the movement feedback and had a sense of self-agency. Thus, ‘0’ was anchored to the congruent situation where the source of the action and its consequences were *uniquely* attributed to the Self. The value ‘5’ was then anchored to the largest possible *external* influence, as manifested by the strongest sense of perceptual-motor conflict, which was demonstrated to them in the pilot trials. The theoretical linear response would then correspond to a linear mapping from the set of angular deviations to the 0 to 5 scale. On this scale, 1 was anchored to indicate the smallest departure from the congruent condition *SR* = 0, i.e. the first subjective experience of perceptual-motor conflict. This means that the participant’s sense of self-agency was disrupted by the *conscious* detection of an external perturbation (angular deviation), and the consequence of acting could no longer be attributed uniquely to self generated action.

At the beginning of an experimental session participants were explained the details of the experiment to get familiarized with the task. Next, they received a training period of at least 10 trials in order to get accustomed to the experimental constraints, experience how external perturbations (angular deviations) were mapped on the visual feedback, and learn to correct the trajectories to optimize their behavioral performance. Training trials were continued until the participants responses were properly calibrated in the range from 0 to 5 in accordance with the possible angular deviations ranging from 0° to ±27°.

Each experimental session consisted of three blocks with 28 trials per block, where the first two blocks included all 56 possible angular biases ([−27°,…, -1°, 0°, 0°, 1°,…,27°], in steps of 1°). Note that both blocks of trials contained the angular deviation 0°. Given that all data were measured twice for the angular deviation 0°, the mean of the two values at 0° was included in the data analysis (see below). Thus, the first two blocks contained 55 trials. In the third block, performance was not affected by an angular deviation. This third block of trials with no angular deviation was included as a control condition for the EEG analysis, so that a comparison between perturbed and unperturbed conditions can be realized. This is necessary for the differential assessment of these two conditions. The order of presentation of the first two blocks was counterbalanced across participants. Here we will present only the data related to these first two blocks.

### Data analysis

Four different dependent variables were obtained from each trial: (i) Error in visuomotor performance, here called motor error (*ME*), (ii) the duration of the movement or movement time (*MT*), (iii) the result of the rating scale also called subjective report (*SR*), (iv) and the angular threshold

As done in previous studies [10-13], ME was defined as the average deviation of the participant’s trajectory from a theoretical line that would perfectly compensate for the bias. *ME* was computed as follows:

(1) $$ME\left(\alpha ,\theta \right)=\sqrt{\frac{1}{N}\sum_{i=1}^{N}{\left(RD_{\alpha ,\theta }^{i}-ID_{\alpha ,\theta }^{i}\right)}^{2}}$$

where: *α*: angular deviation; *θ*: target position angle; *ME (α, θ)*: motor error for a given pair *(α, θ)*.; N: total number of sample points in a trajectory drawn by the pen stylus; *i*: sample number; $RD_{\alpha ,\theta }^{i}-ID_{\alpha ,\theta }^{i}$: actual deviation of the real trajectory (*RD*) from the ideal trajectory (*ID*) for a given pair *(α, θ).*.

The closer *ME* gets towards 0, the better the trajectory matches the desired straight line between the starting point and the target [12].

Movement time (*MT*) was defined as the time span from movement initiation (speed of pen stylus was different from zero) to the moment when participant reached the target (pen stylus is located in the area covered by the target and its speed vanished). We used two criteria to detect when a reaching movement started: i) the position of the green dot should be at least 2 mm away from its initial position (during baseline) within the green square, and ii) The velocity of the green dot should be at least 1.2 cm/s. These two criteria were experimentally found to be appropriate to differentiate between micro-movements of the hand/fingers and a true reaching movement.

An additional analysis of *MT* and *ME* was done to better understand and visualize the effect of mindfulness meditation on visuomotor performance. For the longitudinal design, movement slope (*MS*) was defined as the ratio of change from t1 to t2 of *ME* to *MT,* at each angular deviation *α*. Within each group SM and CG, the value of *MS* was computed as:

(2) $$MS\left(\alpha \right)=\frac{\Delta \bar{ME}}{\Delta \bar{MT}}=\frac{{\bar{ME}}_{t_{2}}\left(\alpha \right)-{\bar{ME}}_{t_{1}}\left(\alpha \right)}{{\bar{MT}}_{t_{2}}\left(\alpha \right)-\bar{MT_{t_{1}}}\left(\alpha \right)}$$

where $\Delta \bar{ME}$ and $\Delta \bar{MT}$ represent the difference in mean motor error and motor time between times t1 and t2. For the cross sectional design, *MS* was defined as the ratio of change between groups NM and LM of *ME* to *MT,* according to the following formulae:

(3) $$MS\left(\alpha \right)=\frac{\Delta \bar{ME}}{\Delta \bar{MT}}=\frac{{\bar{ME}}_{\mathrm{LM}}\left(\alpha \right)-{\bar{ME}}_{\mathrm{NM}}\left(\alpha \right)}{{\bar{MT}}_{\mathrm{LM}}\left(\alpha \right)-{\bar{MT}}_{\mathrm{NM}}\left(\alpha \right)}$$

where $\Delta \bar{ME}$ and $\Delta \bar{MT}$ represent the difference in mean motor error and motor time between the two groups LM and NM at each value of *α*. Note that *MS* is defined both for the ‘within-group’ and the ‘between groups’ comparisons. In both cases *MS* refers to a similar contrast between two conditions, where the main difference relies on the impact of short-term (SM group), long-term meditation experience (LM) or no intervention (CG group) in motor performance.

In a graphical representation of the dependence of *ME* with *MT,* the ratio *MS* would correspond to the slope of the line joining two points corresponding either to times t1 and t2 (longitudinal design) or to groups LM and NM (cross sectional design). In this way, we intend to visualize how the relationship between *ME* and *MT* changes for angles below and above the angular threshold.

The subjective report was recorded on a 6 point scale ranging from 0 to 5. This score was first used to evaluate how participants’ awareness of a perceptual-motor conflict was modulated by the presence of an external perturbation (angular deviation) varying in strength. In addition, this data was taken to compute the angular threshold α* for each participant and each side (left, right). This angular threshold α* indicates the individually-based first experience of perceptual-motor conflict, after which the sense of self-agency is disrupted by the presence of an increasingly perceived external perturbation. *SR* and α* values were obtained for further analysis by performing the following three steps. (i) In order to reduce variations of *SR* and also to obtain the main dependency of *SR* with *α,* a boxcar moving average was applied by computing the mean of *SR* across 5 contiguous α values. Therefore, we had to omit the angles ±26° and ±27° for further analysis since this smoothing procedure excludes these four angles. This procedure reduced the number of trials to 51 ([−25°,…, -1°, 0°, 1°,…,25°]) (ii) When plotting the smoothed SR dependency with α, it was observed that in cases where the angular deviation α was zero or close to zero, some participants failed to recognize the absence or very small effect of the angular deviation. Therefore we anchored all smoothed *SR* data by a linear transformation so that the minimum ratings of the angular deviation *α* for each participant was set to 0. The maximal smoothed *SR* was not affected by this procedure. Since this is a very conservative strategy, it made our analysis robust against the influence of non-specific factors. (iii) In a next step the threshold α* was defined as the first value of *α* after which all transformed values (smoothed and anchored) of *SR* were equal or larger than 1. This definition of the threshold α* is based on the fact that a rating of ‘1’ on the 6-points scale reflects the first individual perception of a perceptual-motor conflict. Note that according to this definition, the average threshold α* across participants is not necessarily the first value of *α* after which all values of the average *SR* are equal or larger than 1.

Consistent with the smoothing procedure applied to the *SR* values, movement time (*MT*) and motor error (*ME*) values were also smoothed following a similar procedure, i.e., a boxcar moving average was applied by computing the mean of *ME* and *MT* across 5 contiguous *α* values.

All calculations were done in MatLab 2007b.

### Statistical analysis

Both comparisons (SM vs. CG and LM vs. NM) were computed by a linear mixed model for each of the three dependent variables ME, MT and SR with the software SPSS 17.0. For the assessment of the MBSR intervention within the longitudinal design the model included the three fixed factors *group* (SM vs. CG), *time* (*t1* vs. *t2*) and *angle* (0° to 25°, 26 levels) as well as the interaction terms *group x time* and *group x angle*. For the cross sectional comparison of long-term meditators (LM) with non meditators (NM) the model included the fixed factors *group* (LM vs. NM) and *angle* (0° to 25°, 26 levels), the interaction term *group x angle* as well as the covariate *age*. For the assessment of the fourth dependent variable, i.e. the angular threshold *α*, we performed a repeated measurement ANOVA (factors *group* and *time*) for the longitudinal and an ANCOVA (factor *group*, covariate *age*) for the cross sectional part of the study. Note that the angular threshold is based on an evaluation of all angles and thus the factor angle cannot be computed. For the differences in angular thresholds the effect size Cohen’s d was calculated. The exact syntax can be obtained from the authors.

## Results

### Self-attribution of mindfulness

The results of the Freiburg Mindfulness Inventory measuring self-attribution of mindfulness consistently showed that practicing mindfulness meditation is associated with a higher level of self-attribution of mindfulness. Short-term meditators (SM) showed a significant increase in self-attribution of mindfulness (*t* = 6.16, *df* = 10, *p* < .001). A repeated measurement ANOVA with the factor time (t1, t2) and group (SM, CG) showed as expected a significant *time x group* interaction (*F* = 13,67, *df* = 1, *p* = .02). Also long-term meditators showed a score significantly different from the NM group (*t* = 7.44, *df* = 18, *p* < .001). Thus the questionnaire data support also empirically the justification of the comparisons made in the two parts of this study.

### Effect of side of perturbation on *ME*, *MT*, *SR* and α*

In order to see whether the data recorded from left- and right-biased trials (i.e. perturbed by an angular deviation towards either the left or the right side) can be combined, we performed at first significance tests comparing right vs. left for all dependent variables and measurement points. Raw data and inference statistics can be seen in Table 1 below. There are significant differences for the main effect of side of angular perturbation in all dependent variables at *t1* and in two out of four variables at *t2*. In fact, angular perturbations to the right led to larger *ME* and larger *MT,* larger *SR and* smaller angular threshold α*.

**Table 1:** Comparison of side of perturbation (left or right) for both measurement points t1 and t2

***Variable***		***Mean (SD)***	***Mean (SD)***	***p***
		***left***	***right***	
*ME (mm)*	*t1*	4.00 (2.07)	5.38 (1.59)	0.001
	*t2*	3.68 (1.25)	5.02 (1.51)	<0.001
*MT (ms)*	*t1*	5619 (1906)	5860 (1900)	0.003
	*t2*	6221 (1824)	6386 (1912)	0.08
*SR*	*t1*	1.64 (0.67)	2.17 (0.59)	0.001
	*t2*	1.59 (0.70)	2.16 (0.68)	0.002
α*(°)	*t1*	13.68 (5.86)	7.71 (4.48)	<0.001
	*t2*	10.18 (4.43)	8.23 (3.60)	0.14

Means were calculated over all 25 angles to the left and the right respectively, p-values are based on paired sample t-tests. For t1 all analyses are based on N = 31, for t2 N = 22. ME = motor error, MT = movement time, SR = subjective report, α* = angular threshold.

The difference between right and left side is most likely due to an incongruence associated with the fact that the target always appeared in the right visual field and the task had to be performed with the right hand. If the angular deviation is applied to the left (which requires a compensatory movement to the right), it results in a congruent condition facilitating the task. By the contrary, an angular deviation to the right entails a movement to the left, which accordingly triggers a mismatch between movement direction and target position, likely disturbing visuomotor performance. Thus further analysis will be based on the congruent left side only (26 angles in the range [−25°,…,-1°,0°]), since this will lead to more accurate data regarding the effects of meditation practice (for more details see discussion).

### Longitudinal comparison: assessment of the MBSR intervention

Table 2 reports the results of the longitudinal comparison (SM & CG).

**Table 2:** Results of the mixed linear model analysis and the repeated measurement ANOVA for the four dependent variables

***Variable***	**ME**		**MT**		**SR**		α*	
	***F*(*df*)**	***p***	***F*(*df*)**	***p***	***F*(*df*)**	***p***	***F*(*df*)**	***p***
*group*	106.20	<0.001	73.5	<0.001	0.003	0.958	0.001	0.98
	(1/623.0)		(1/1051.9)		(1/809.4)		(1/20)	
*time*	5.68	0.02	40.23	<0.001	8.01	0.005	3.95	0.061
	(1/577.0)		(1/1055.2)		(1/557.9)		(1/20)	
*angle*	10.93	<0.001	1.34	0.16	112.36	<0.001	-	-
	(25/75.2)		(25/84.2)		(25/88.2)			
*group x time*	27.60	<0.001	9.64	0.002	14.05	<0.001	1.60	0.22
	(1/577.0)		(1/1055.2)		(1/557.9)		(1/20)	
*group x angle*	1.87	0.02	0.04	1.00	0.58	0.941	-	-
	(25/75.2)		25/84.2		(25/88.2)			

ME = motor error, MT = movement time, SR = subjective report, α* = angular threshold.

### Subjective report

**Figure 3:**
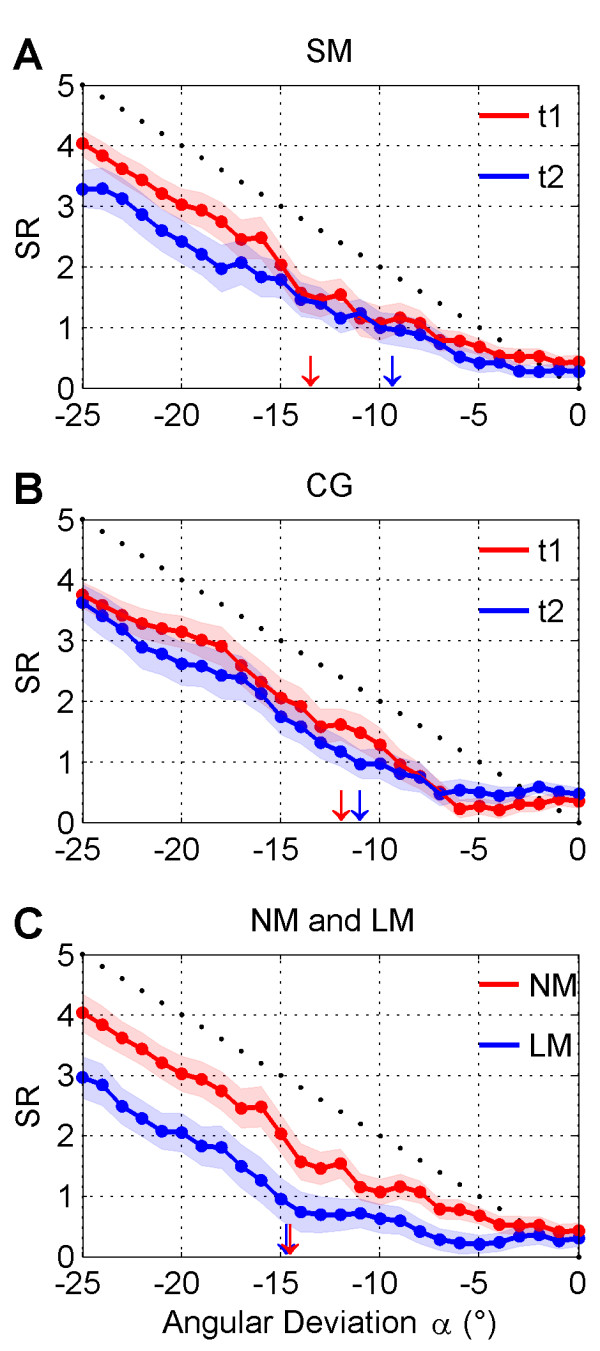
**Mean and Standard Error of Subjective Report (SR). A**) Comparison between SR values before (t1) and after (t2) the MBSR intervention for the SM group. **B**) Comparison of SR values at times t1 and t2 for the CG group. **C**) Comparison of SR values between the NM (red) and LM (blue) groups. Shading indicates standard error (SE). SR data for t1 and t2 measurement points and groups LM and NM are denoted by blue and red colors respectively. Downward arrows indicate the adjusted mean of angular threshold in each group and measurement point (same color coding).

The subjective report of the participants showed as expected a main effect for *angle* (p < .001). As shown in Figure 3A and 3B, subjective report was increasing for larger angular deviation, and kept below the theoretical linear response for both groups SM and CG and at times t1 and t2. There was also a main effect for *time* (*p* = .005) with the adjusted means of *SR* dropping from 1.73 at *t1* to 1.63 at *t2*. Lower *SR* values at t2 were observed mostly for deviation angles above the angular threshold at t2 (*cf.* Figure 3A and 3B). The *group x time* interaction reflecting the effect of the intervention was also highly significant (p < .001). The adjusted means showed that *SR* values in the intervention group SM dropped from 1.80 at t1 to 1.56 at t2. At the same time the control group (CG) scored 1.67 and 1.70 respectively. As seen in Figure 3A, the tendency to reduce the rating of perceptual-motor conflict was much stronger in the SM group after the MBSR intervention (t2), and for angular deviations larger than the threshold α*.

### Angular threshold

We found no significant effects for the factor *group*, and the *group x time* interaction (p = .22), but a close to significant effect for the factor *time* (p = .06). Interestingly, this effect of time was more prominent in the SM group, where we obtained, in accordance with our hypothesis, a decrease in angular threshold from −13.45° (SD = 6.90°) at t1 to −9.36° (SD = 5.54°) after the MBSR intervention (*t2*) resulting in a medium effect size of *d* = 0.65 (see arrows in Figure 3A). Although in the CG group the angular threshold was lower at baseline (t1), changes from t1 to t2 were minimal, from −11.91° (SD = 5.30°) to −11.0° (SD = 3.0°) with *d* = 0.21 (*cf.* Figure 3B). Thus, the MBSR intervention resulted in an earlier detection of perceptual-motor conflict at the fringe of awareness, which in turn led to a departure from self-attribution of agency at lower levels of external perturbation.

**Figure 4:**
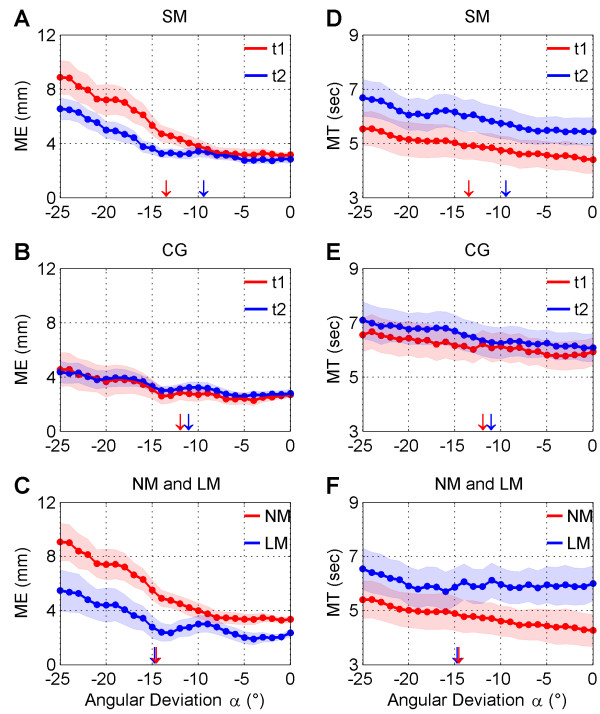
**Mean and Standard Error of motor error (ME) and movement time (MT).** ME and MT data are separately represented in the left panel and right panel respectively. A-D) Comparison of ME and MT values before and after the MBSR intervention. B-E) Comparison of ME and MT average at times t1 and t2 in the CG group. C-F) ME and MT are represented for the comparison of the LM and NM groups respectively. Shading indicates standard error (SE). Data for t1 and t2 measurement points and groups LM and NM are denoted by blue and red colors respectively. Downward arrows indicate the adjusted mean of angular threshold in each group and measurement point (same color coding).

### Motor error

As can be seen in Table 2, there were significant main effects for all three factors *group, time* and *angle*. As expected, motor error increased with angular deviation for both groups (*cf.* Figure 4A and 4B). The effect of the MBSR intervention is reflected by the highly significant *group x time* interaction (*p* < .001). This effect was mainly due to an improvement of the SM group. An inspection of the adjusted means show that the visuomotor performance in the SM group improved after the MBSR intervention, i.e. *ME* decreased from 4.71 mm at time t1 to 4.08 mm at t2. As seen in Figure 4A, the reduction of *ME* at t2 in the SM group was obtained mainly for angles above the angular threshold at t2. The control group had overall a better visuomotor performance (i.e. smaller ME) but did not show an improvement from t1 to t2. There was a non-significant decrease in performance from t1 (ME = 3.11 mm) to t2 (ME = 3.34 mm).

### Movement time

For this variable we obtained significant main effects for the two factors *group* and *time*. Participants showed a baseline difference in MT, i.e. reaching movements were faster in the SM than in the CG group. Although we found a slight tendency for an increase of *MT* with angular deviation (*cf.* Figure 4D and 4E), no significant effect of the factor angle was obtained. The relevant interaction *group x time* was highly significant (*p* = .002). This effect was mainly due to the intervention group SM (*cf.* Figure 4D), which slowed down by approximately 1 second (MT = 4901 ms at t1, MT = 5902 ms at t2) while the control group, although significantly slower (MT = 6151 ms at *t1*, MT = 6494 ms at *t2*), showed a smaller increase of 343 ms only. Contrary to the SM group, this small increase in movement time was not associated with lower ME in the CG group.

### Movement slope

**Figure 5:**
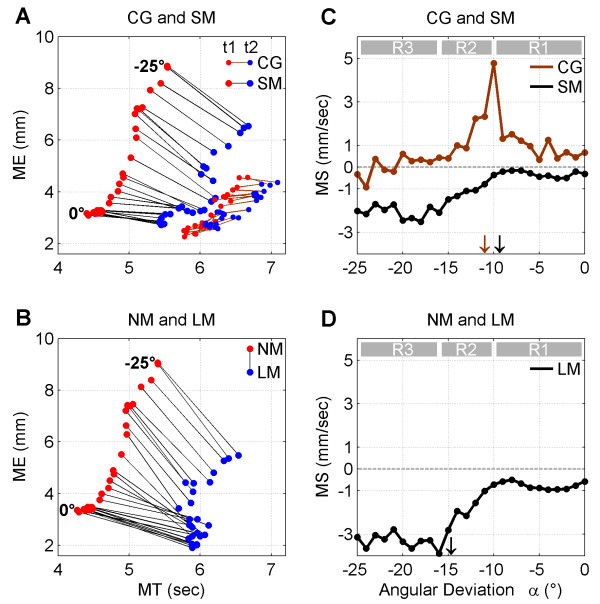
**Movement slope (MS). A**) Comparison of adjusted means of *ME* and *MT* at each angular deviation *α* for both measurement points (t1 and t2) and both groups (CG and SM). For each *α* value, data points for t1 and t2 are connected by a black line within each group SM and CG. **B**) Comparison of *ME* and *MT* at each angular deviation *α* for both groups NM and LM. Data points for each *α* value are connected by a black line between the two groups NM and LM. In A) and B), the dependence of *MS* with *α* can be visually estimated by the change of steepness or slope across the lines. The pairs (*MT, ME)* for times t1 and t2 and groups LM and NM are denoted by blue and red colors respectively. **C**) Dependence of *MS* with *α* for both CG (brown) and SM (black) groups. Downward arrows indicate the adjusted mean of angular threshold at time t2 for both groups (similar color coding). **D**) Dependence of *MS* with *α* for the comparison NM & LM. The downward black arrow indicates the adjusted mean of angular threshold for the LM group. The three *α*-ranges R1, R2, and R3 are represented in gray.

Figure 5A and 5B provide a compact representation of how the values for *ME* and *MT* change for the three factors *group* (CG and SM), angle (0°-25°), and *time* (t1 and t2). A different visualization of the dependence of *MS* with *α* is given in Figure 5C and 5D, where three *α*-ranges R1 (0°, -10°), R2 (−10°, -16°), and R3 (−16°,-25°) were defined.

For the SM group one can observe an increase in *MT* (i.e., lower movement speed) from t1 to t2 that is at the same time associated with an improvement in performance, i.e. a decrease in ME (*cf.* Figure 5A). Therefore, from t1 to t2 participants behavior changed from a ‘fast and less accurate’ to a ‘slow and more accurate’ behavioral strategy. These transition resulted in a negative movement slope (*MS*), as seen by the steepness of the lines connecting the pairs (*MT*,*ME*) at times t1 and t2 for each value of *α*. Note that *MS* (slope of the lines) change continuously from 0° to −25°. By the contrary, in the CG group changes from t1 to t2 in *ME* and *MT* were smaller as represented by shorter lines, and resulted in mostly positive values of *MS.*

As seen in Figure 5C, the two groups showed a clear difference regarding their change in behavioral strategy between t1 and t2. For the CG group, *MS* values were mostly above 0, reflecting that there was no overall improvement in performance at t2 (see also Figure 4B). For angles below the angular threshold at t2 (R1), *MS* increased up to ~5 mm/sec at *α* = 10°, meaning that although *MT* increased from t1 to t2, *ME* also increased up to 5 times more than *MT*. In the range of *α* values above the angular threshold R2, *MS* steeply decreased but remained above 0, as there was no improvement in performance. For R3 *MS* decreased slowly and turned negative (better performance at t2) only for the largest angular deviations.

For the SM group, *MS* values resembled a sigmoid-like function and were negative for the whole range of angular deviations, meaning that an increase in *MT* was associated to improved performance. For angles below the angular threshold at t2 (R1), *MS* values were approximately constant and above −1 mm/sec, meaning that *ME* decreased at t2, but in lower proportion to what *MT* increased. For *α* values above the angular threshold at t2 (R2), *MS* values decreased monotonically down to values around −2 mm/sec. Thus, in R2 performance improved after the MBSR intervention at increasing rate compared to *MT*. This effect is also clearly observed in Figure 4A and 4D. In R3, *MS* values stabilized around −2 mm/sec.

### Cross-sectional comparison: assessment of long-term meditation effects

In the cross sectional analyses the performance of 9 long-term meditators was compared to the performance of 11 novices to meditation. We entered age as a covariate in the model since the long-term meditators were of different age than the controls. It could be shown that age had a significant influence on the behavioral performance in our task. Table 3 reports all results of the cross-sectional comparison.

**Table 3:** Results of the mixed linear model analysis and the repeated measurement ANCOVA for the four dependent variables

***Variable***	**ME**		**MT**		**SR**		α*	
	***F (df)***	***p***	***F (df)***	***p***	***F (df)***	***p***	***F (df)***	***p***
*group*	77.94	<0.001	37.09	<0.001	71.99	<0.001	0.00	0.99
	(1/284.4)		(1/463.8)		(1/409.9)		(1/17)	
*angle*	6.63	<0.001	0.26	1.00	35.35	<0.001	-	-
	(25/42.3)		(25/35.2)		(25/38.4)			
*group x angle*	0.82	0.70	0.09	1.00	1.39	0.18	-	-
	(25/42.3)		(25/35.2)		(25/38.4)			
*age (cov.)*	45.19	<0.001	11.38	0.001	6.29	0.013	4.43	0.051
	(1/144.1)		(1/457.9)		(1/314.3)		(1/17)	

Age is entered into the model as covariate. ME = motor error, MT = movement time, SR = subjective report, α* = angular threshold, cov. = covariate.

### Subjective report

As seen in Table 3, we found a highly significant main effect for angle (p < .001). Average ratings increased for larger angular deviation, but both groups, LM and NM, were below the theoretical linear response (*cf.* Figure 3C). Remarkably, in the LM group the *SR* increase with *α* speeded up for angles above the angular threshold. We also found a highly significant main effect for group (p < .001). An inspection of the adjusted means showed that long-term meditators had much lower average of subjective ratings of 1.15 compared to 1.80 for non-mediators. This was especially true for external perturbations above the threshold (*cf.* Figure 3C). If this finding is compared to the longitudinal part of our study, one can see in Figure 3A that a similar meditation-related reduction of the subjective rating of perceptual-motor conflict was also observed in the SM group.

### Angular threshold

The comparison of the angular threshold between these two groups was performed by a simple ANCOVA with age as covariate. There was no significant difference between the groups. The α* adjusted means were almost identical (−14.66° and −14.64° for the LM and NM groups respectively). This result did not match the meditation-related tendency to detect earlier a mismatch between perceptual and motor information as observed in the SM group (see Figure 3A).

### Motor error

Table 3 shows the main effects and the relevant interactions of the linear mixed model. There was a highly significant main effect for the factors *angle* and *group* (*p* < .001). As expected, larger angles led to higher difficulties to compensate for external perturbation and hence to larger *ME* (*cf.* Figure 4C). Long-term meditators were much more accurate in their performance. They had a mean motor error of 3.24 mm compared to 5.36 mm in non-meditators. For the LM group, mean *ME* values were kept low for angles below the angular threshold. For angles above the angular threshold, *ME* monotonically increased with angle, but remained lower than *ME* values in the NM group. A similar meditation-related improvement in visuomotor control was found in the SM group (*cf.* Figure 4A).

### Movement time

In Table 3 it is shown that there is a highly significant main effect for *group* differentiating between long-term meditators (LM) and non-meditators (NM) (*p* < .001). The adjusted means demonstrate that long-term meditators have a much slower performance with an average of 5985 ms compared to 4766 ms in non meditators. Strikingly, *MT* values in the LM group remained approximately constant for most angles, increasing only for large angles (*α* ≥ 20°). In the longitudinal study the short-term meditator group (SM) also showed a corresponding slowing-down in *MT* which can be associated to meditation practice (MBSR intervention) (*cf.* Figure 4D).

### Movement slope

As seen in Figure 5B, the LM group followed a strategy of slower and better performance than the NM group (see also Figure 4C and 4F) which performed the task faster with corresponding larger motor errors *ME*. This difference in strategy resulted in a negative slope (*MS*) of the lines connecting the pairs (*MT*,*ME*) of the two groups at each value of *α*. Note that the steepness of the lines (*MS* values) decreased almost continuously from 0° to −25°. This effect is better observed in Figure 5D. Strikingly similar to the SM group (*cf.* Figure 5C) in the longitudinal comparison, *MS* values followed a sigmoid-like function and were negative for the whole range of angular deviations. In R1, *MS* values were approximately constant and above −1 mm/sec. As seen in Figure 4F, *MT* increased with *α* in the NM group, while in the LM group *MT* values were much larger and remained approximately constant in R2. In addition, while *MT* values did not increase in the LM group, *ME* values decreased with *α* (*cf.* Figure 4C). The combined effect of these changes in *MT* and *ME* resulted in a monotonically decrease of *MS* values down to −4 mm/sec, i.e. twice the size compared to the *MS* values of the SM group in the longitudinal study (*cf.* Figure 5C). In R3, *MS* values stabilized around −3 mm/sec.

## Discussion

The present study was designed to investigate if mindfulness meditation modulates perceptual-motor awareness, self-agency and visuomotor performance. Overall, our results demonstrate that experience with mindfulness meditation leads to a change in movement experience and to a different behavioral strategy, where performance accuracy is improved primarily due to the practice of mindfulness meditation, and is concurrent with slower task execution. Furthermore, we also found that practice of mindfulness in the context of an MBSR intervention resulted in a lowered threshold for the detection of a perceptual-motor conflict. Therefore, novices in meditation experienced a disruption of the sense of self-agency at lower levels of external perturbation after the meditation training compared to controls. This finding was not true for long-term meditators. On the other hand, the size of the perceptual-motor conflict was underrated by both novices and long-term meditators for external perturbations above the detection threshold. We will separately discuss the different aspects of these results below.

### Effects of direction of angular deviation

We observed that an angular deviation to the left resulted in a more congruent condition facilitating the task while an angular deviation to the right is likely impaired by an incongruent movement. This asymmetry is probably associated with differences in task difficulty for the two sides. When the trajectories are perturbed toward the left, participants had to compensate for the distortion with a deviation of the hand direction *towards the right*. As the target appeared always on the right visual field, movements were performed with the right hand, and hand direction was also towards the right, this congruency of location and direction of movement might have facilitated the task, and thus lead to lower motor errors and shorter movement times. In contrast, the incongruence between target location (right) and direction of movement (left) for trials with an angular deviation towards the right may have lead to larger difficulties in performing the task, and therefore larger motor errors and larger movement times. Similar congruency effects of target location, hand and movement direction has been reported for reaching movements elsewhere [42,43]. Due to the incongruence of side and task direction we restricted our analyses to the congruent trials only (left side).

### Effects of mindfulness on movement time and visuomotor performance

When comparing short-term meditators (SM) with non-meditators before and after the MBSR intervention, we observed that the SM showed a reduction in motor errors and this improvement can be attributed to the intervention. Additionally, while the participants improved in accuracy at post-test (t2) they showed significantly larger movement times (*MT*) than at baseline (t1). The requirement in aimed movements of increasing movement time in order to achieve better performance (lower motor error) has been also referred as speed/accuracy trade-off [44]. This raises an important question: is the observed improvement in motor performance merely the consequence of slowing down, or primarily triggered by additional cognitive factors associated to the practice of mindfulness meditation.

Both, lower motor errors and larger movement times can be related to the MBSR intervention. This finding indicates that the mindfulness intervention lead to slower reaching movements and likely to a corresponding slowing down of the behavioral tempo. This finding is confirmed by similar results from the cross sectional study. Here we also found significant lower motor errors in long-term meditators compared to non-meditators. In addition, better performance in the LM group was accompanied by a significant larger movement time compared to the NM group. Both effects on movement speed and motor accuracy were much stronger for deviation angles above the threshold, i.e. when participants were aware of the external perturbation, and thus were (mindfully) monitoring their movements and consciously implemented a behavioral strategy to compensate for the external bias. These concomitant changes associated to movement time and motor error can be clearly observed in Figure 5. The movement slope (*MS*) showed that short- and long-term meditators followed similar strategies while performing the task. Namely, they significantly slowed down their movements, and were able to decrease motor error. When they were unaware of the external perturbation, the gain in accuracy was smaller than the increase in movement time (−1 < = *MS* < = 0). Interestingly, *MS* decreased dramatically below −1 according to a sigmoid-like function once meditators detected the bias, meaning that the gain in accuracy was much larger for similar amounts of increase in movement time. This suggests that the increase in motor accuracy cannot be explained out solely by a concomitant increase in movement time. As can be seen in Figure 5C and 5D long-term meditators gain in accuracy (*MS* = −4) was approximately twice as much as in the SM group (*MS* = −2), suggesting that becoming aware of the external perturbation had a stronger impact in the behavioral performance in long-term meditators. In the face of an increasing perturbation above 16°, both meditation groups were unable to decrease further their *MS* values but remained fluctuating around constant values. This suggests that *α* = 16° could be interpreted as a second angular threshold, after which the external perturbations got in balance with the compensatory efforts of the subjects. Compared to the SM group, non-meditators performed the task at a much higher tempo, and showed a different strategy in compensating for the external perturbation. This is in line with other studies of movement speed during reaching at different paces [45]. We would like to emphasize that the abrupt decrease in *MS* after an angular deviation ~ −10° in both t2 vs t1 (SM group) and LM vs NM comparisons, implies that there was additional reduction of motor errors that cannot be explained out by a linear increase of movement time. We take this non linearity (i.e. increasing rate of motor control improvement with movement time) as an indication that the practice of mindfulness meditation has a direct positive effect on motor accuracy that goes beyond a mere slowing down of the actions. By the contrary, mindfulness seems to dynamically modulate the speed/accuracy trade off governing the relationship between movement time and motor errors. All together, the results of our study suggest that mindful movements could be reliably associated with higher motor accuracy, slower speed, and the relationship between them, resembling the dynamic operation of a speed/accuracy trade-off principle.

In many types of perceptual-motor tasks, a trade-off between movement speed and performance accuracy has been already found (see [44] for a comprehensive review). That is, a participant can either perform the task very fast with many errors or very slow with only few errors. Participants in this study were constrained to a time range of 3 – 15 sec. (i.e. *MT*), but they were free to choose the strategy that best suited their individual tempo and which was still congruent with the request for optimal accuracy, i.e. to minimize trajectory deviations. Our data demonstrate that short- and long-term meditators achieved higher motor accuracy while slowing down their movements, if they were asked to act mindfully. The slow speed likely gave them time to better monitor *moment-by-moment* the quality of their performance. This strategy is compatible with less automatism in motor control and likely with larger ‘top-down’ cognitive regulation of the sensorimotor integration processes. As stated above, we hypothesize that these additional cognitive factors played a major role on the improvement in motor control beyond the positive effects of slowing down the movements.

### Effects of meditation on self-agency

We found a pronounced reduction of the angular threshold in the hypothesized direction in the short-term meditators (d = 0.65) after the intervention but only a small decrease in the control group (d = 0.21). The most likely interpretation of this finding is that by virtue of mental training in attentional regulation and meta-awareness associated to the MBSR intervention, short-term meditators had a wider access to the varieties of sensorimotor and proprioceptive signals necessary to evaluate the perceptual-motor conflict at the fringe of detection. Thus, novices may have gained a finer discrimination of self-agency disrupting signals at lower levels of external perturbation. Nevertheless, this interpretation should be taken cautiously since this finding failed to reach significance. This is most likely due to the low statistical power of this specific analysis. Unlike for the other variables reported in this study there is only one threshold value per participant and measurement time. In order to turn a difference of this size significant a larger sample is needed. In addition, the comparison for the long-term meditators with their respective controls showed no such difference. One possibility for this mismatch is that long-term meditators have interpreted the task in a different way compared to novices and non-meditators. We instructed all participants to give a rating of *SR* = 1 for the first experience of a perceptual-motor conflict. If the “mindful” experience of a moving limb entails a wider access to the set of sensorimotor and proprioceptive signals arising from the body, it is plausible that long-term meditators, although being aware of an external perturbation at lower angles, did not interpreted this event as a disruption of the sense of self-agency. Buddhist meditators are known to cultivate an unorthodox attitude towards the self (*theory of anatta*) , which neglects a narrative based self-concept [22,46,47]. It is likely that this results in a trait change to the frontoparietal network underlying action monitoring [48-50], and thus might result in a recalibration of the neural comparators underlying the conscious detection of perceptual-motor conflict at the fringe of awareness. This explanation finds support in two empirical findings: i) *MS* values started to decrease steeply around *α* = 8° (see Figure 5D) in the LM group (similar to the SM group), suggesting that this was triggered by the conscious detection of an external influence. ii) As can be seen in Figure 4C and 4F, above *α* = 8° *MT* remained constant and ME even decreased. On the contrary, above the reported angular threshold of *α* = ~15°, motor error and movement times started to increase sharply with *α.* Thus, these data suggest that LM meditators rather adjusted an inner perspective while crossing the threshold of detection and only reported a disruption of self-agency when angular deviations were very large. At such a point compensation was much more difficult, leading to a larger mismatch between visual feedback and intentions. This purported recalibration process may also explain the reduction of *SR* values in novices and experts in meditation for angles in the range R2 and R3, as discussed below. However, it has to be noted that this interpretation remains to some point speculative. As an explanation of an exploratory finding it should be at first confirmed in a new investigation.

### Effects of meditation on the detection of a perceptual-motor conflict

Two variables are reflecting the effects regarding the detection of a perceptual-motor conflict. One is the angular threshold α* and the other one is the subjective report (*SR*). *SR* was significantly reduced by the MBSR intervention (group x time effect) and the inspection of the means indicated that *SR* values decreased in the intervention group while it remained almost unchanged in the control group. This finding is replicated by the results of the long term meditators. They showed the same tendency and also scored significantly lower than their respective controls. A reduction in *SR* means that in the assessment of a given external perturbation the subjective impact of this perturbation is reduced or in other words the same perturbation is more likely attributed to an internal process than to an external influence.

Such a finding of a change in attribution towards internal processes seems to be a contradiction to the lowered thresholds which were also related to meditation experience (see above). One likely interpretation for this reduction in the experience of perceptual-motor conflict may be that it is due to the explicit attention to the own body movements associated with mindfulness practice, i.e. the conscious observation of the movements. One can assume that the training and practice in mindfulness results in a pronounced shift in attention and awareness while exerting the movement. While untrained or control subjects will pay more attention to the target to reach and the visual feedback, trained meditators will most likely pay more attention towards the varieties of sensorimotor and proprioceptive signals elicited by the process of the movement itself as this is taught in mindfulness moving. This process will be additionally boosted by the fact that the practice of mindfulness leads to significant slower movements in both meditation groups. But such a conscious focus on sensorimotor and proprioceptive signals will most likely result in a shift of the evaluation of the perceptual-motor conflict towards internal processes.

If such an effect shows up irrespective of the strength of the external influence then it would result in a more inaccurate overall judgment since the threshold of detection of perceptual-motor conflict would be shifted towards a higher value. But the opposite is true and an inspection of Figure 3A gives some clues why this is the case. First of all this effect of a reduction in *SR* due to meditation experience is almost only present for the larger angles above the threshold at t2 (*α* > 15°). In this case the presence of an external influence is obvious and the subjective rating is giving a judgment of the relationship between external and internal influences. In the area around the threshold (9° to 15°) the two curves are much closer to each other. The threshold is mathematically determined by an algorithm which is build in order to give a conservative estimate and to avoid misjudgments by relying on single successful trials which may be due to chance. The application of this procedure results for the intervention group in a strong reduction of the threshold by more than 4°. This reduction shows that the participants are much more sensitive to the external influences in trials where they are difficult to detect. By this interpretation the effects of a shift towards reduced detection of perceptual-motor conflict for higher angles and a better sensitivity to detect the external manipulation for lower angles are not contradictory to each other. However the latter finding is not confirmed in the long-term meditation group. This underlines the interpretation that there are two distinct processes at work. One is related to the subjective evaluation of self-agency (detection threshold) and the other to the estimation of the size of the external perturbation that leads to the generation of a perceptual-motor conflict. This kind of differential effect has been previously found in the context of perceptual-motor conflict and a similar dual-process interpretation has been advanced [51].

### Methodological issues of this study

The study of the influence of mental training on behavior and cognition is associated with non-trivial methodological challenges, especially if dealing with the multiple factors that may underlie meditation training effects (see [52] for a comprehensive discussion). To assess the above mentioned effects of mindfulness meditation, our study consisted of two different parts (cross-sectional and longitudinal) with two different groups of meditators. These were novices in meditation and practitioners with extremely long-standing meditation experience. We explicitly chose this combined design in order to overcome shortcomings of each of the two separate types of studies. The study of long-term meditators provides an excellent experience within the meditation task but lack a good control condition. Differences to a matched non-meditating control group can never be causally attributed to the meditation practice as this is possible in longitudinal studies. Such causal inference is the advantage of working with novices in a controlled longitudinal design. On the other hand in these intervention designs the question remains open whether observed effects are really due to the practice of meditation. They may be also resulting from other non-specific aspects of the intervention e.g. the social interaction within the intervention group. Furthermore in novices it is often unclear whether effects are due to the meditation itself or due to the effort to learn meditation. These two aspects are tightly entangled and can only be separated by a replication of the same findings also in long-term meditators. In contrast to novices it can be assumed for long-term meditators that the efforts to learn and maintain a meditation state are minimized. With our combination of these two research designs we are able to get best possible conclusions regarding the effects of mindfulness meditation to the variables under scrutiny.

## Conclusion

Our investigation identifies, for the first time to our knowledge, *behavioral signatures of mindfulness* in the context of a perceptual-motor integration task. Our results demonstrate that the practice of mindfulness meditation either in a spiritual context, or operationalized by a secular intervention (MBSR), is associated with *slower body movements* under demanding events from the environment and to a larger extent with *reduced movement errors*. On the basis of our data we suggest that in the context of a perceptual-motor task, where subjects were asked to reach a target, this behavioral shift towards an ‘expanded tempo’ may facilitate a broader access to sensorimotor and proprioceptive signals which are crucial for motor control in the face of perceptual-motor conflict. As a consequence of this ‘inner openness’ to the movement experience, a moment-by-moment monitoring of body states could be realized and online re-adjustment of the movement trajectory is optimized. The final result of this cognitive rearrangement leads to an improvement of motor performance and to optimized reaction to the perceptual-motor conflict. At the same time we could also demonstrate that novices in meditation improved strongly in their capability to detect subtle external influences as reflected by a pronounced albeit not significant reduction of the respective threshold of detection.

Our present work embodies a unique contribution by providing for the first time data on the impact of mindfulness meditation on visuomotor performance, perceptual-motor awareness and the sense of self-agency.

## Abbreviations

ANOVA: Analysis of variances; CG: Control group of non-meditators; DT: Digitizing tablet; LM: Group of long-term meditators; NM: Group of non-meditators (with no meditation experience); MBSR: Mindfulness based stress reduction; ME: Motor error; MT: Movement time; PT: Projection tablet; SM: Group of short-term meditators (receiving a MBSR intervention); SR: Subjective report; *α*: angular deviation; *θ*: target position angle; *ME (α, θ)*: motor error for a given pair *(α, θ)*.; N: total number of sample points in a trajectory drawn by the pen stylus; *i*: sample number; $RD_{\alpha ,\theta }^{i}-ID_{\alpha ,\theta }^{i}$: actual deviation of the real trajectory (*RD*) from the ideal trajectory (*ID*) for a given pair *(α, θ).*.

## Authors’ contributions

Conceived and designed the experiments: JRN and SS. Performed all subjects’ recruitment and psychometric assessments, as well as management of the MBSR intervention: SS. Performed the experiments: JRN. Analyzed the data: JRN. Statistical evaluation of the results: SS. Wrote the paper: JRN and SS. Both authors read and approved the final manuscript.
